# Experiencing motherhood as a blind mother in the Greater Accra Region of Ghana; a qualitative study

**DOI:** 10.1186/s12884-022-05153-5

**Published:** 2022-12-04

**Authors:** Angela Kwartemaa Acheampong, Margaret Marfo, Lydia Aziato

**Affiliations:** 1 School of Nursing and Midwifery, Wisconsin International University College-Ghana, Accra, Ghana; 2 University of Health and Allied Sciences, Ho, Ghana

**Keywords:** Ghana, Blind mothers, Mothering with disability, Qualitative research

## Abstract

**Background:**

Females in developing countries are likely to suffer from visual impairment more than their male counterparts. People living with blindness or any other form of disability also have the right to reproduce and care for their children like all other humans. There is paucity of literature in the experiences of blind mothers in Ghana. Therefore, this study explored the experiences of blind mothers as they navigated the process of motherhood.

**Methods:**

Qualitative exploratory descriptive design was used to conduct the study. Nineteen blind mothers were interviewed individually. Permission was sought for data to be audiotaped, transcribed and content analyzed inductively.

**Results:**

Three main themes emerged from the data: mothering role and difficulties (sub-themes; feeding, disciplining and protection), mothering challenges (sub-themes; discrimination and prejudice, financial distress and psychological distress) and coping strategies (prayer, children and self-motivation). Most of the mothers reported difficulties in playing their roles in the areas of feeding, disciplining and protection. Challenges were poverty, discrimination, prejudices on their ability to be mothers and psychological distresses such as depression. They coped with their challenges with prayers, self motivation and the hope they had in their children.

**Conclusion:**

A lot of public education is needed to make the lives of people living with disability better. Health professionals must be trained to treat blind mothers with dignity and respect.

## Background

Globally, the population of people with various types of visual impairments is at least 2.2 billion out of which 1 billion cases could have been prevented [1]. Nineteen percent (19%) of the world’s population of women live with some form of disability [2]. Visual impairment cases in developing countries are four times higher than that of those in developed countries [1]. Visual impairment can create a deficit in the capacity of persons to carry out activities of daily living, hinder their quality of lives as well as limit their ability to socialize with their immediate environment [3]. According to the American Academy of Ophthalmology, women are at greater risk of visual loss than men [4]. The higher prevalent rate of blindness among women across the world is as a result of the higher life expectancy among women and the inadequate access to health care services in low income countries [5]. Blindness among women can be prevented by early detection and prompt treatment [6]. Out of the current Ghanaian population, approximately 8% (representing 2,098,138) have some form of disability. Higher proportions of disability have been recorded in females (accounting for 8.8%) compared to males (representing 6.7%) [7]. In Ghana, women living with visual impairments in their childbearing age account for 11. 1% of the entire female blind population [7].

Persons with disabilities also have the rights to enjoy sexual and reproductive rights just like all other humans [8]. Motherhood is mostly a wonderful experience where women learn to form a sense of self and new identities as they integrate their new born children into their lives [9–12]. The blind mother also goes through birth and breastfeeds their newborn similar to any other mother without disability [13]. Nevertheless, as women with disabilities look forward to this beautiful experience of motherhood, they are scorned, abused, discriminated, stigmatized and face some forms of injustices [14–18]. The discriminations against women with all forms of disability including blindness limit them from exercising their full sexual and reproductive rights as women [19–23]. Article six (6) of the Convention of the Rights of People Living with Disabilities states that “Parties should recognize that women and girls with disabilities are subject to multiple discrimination, and therefore, all appropriate measures should be taken to ensure the full development, advancement and empowerment of women, for the purpose of guaranteeing them the exercise and enjoyment of the human rights and fundamental freedoms set out in the present Convention” [24].

Findings from a study to explore the barriers to marriage and motherhood: the experiences of disabled women in Malaysia espoused that, women with disabilities are very often told that they are incapable of experiencing motherhood [25]. It is even more intriguing according to a study that the immediate family members who are supposed to be the core social support system question the appropriateness of a woman who is blind as a mother [26]. They raise concerns about the possibility of these mothers transferring the disability trait to their children and sometimes suggest abortions to them when they get pregnant [26–28]. In low and middle-income countries the bizarre treatment family members and the community at large subject mothers with disabilities to leave them with low self-esteem and lack of confidence in their motherhood abilities [29].

On the other hand, some women with disability have dared all odds to overcome some forms of stereotypical behaviors the society holds about them to become pregnant, go through normal delivery and raise their children as any other mother does [30, 31]. Such mothers are able to adapt specialized skills and employ alternate senses to provide physical care, comforting measures, engage in play activities, protect and love their children with the help of their spouses and their mothers [16, 32], but among blind mothers, much concern has been expressed about achieving optimum communication between them and their children [33]. However, another study illuminated that, it is more challenging for mothers with disabilities to bath, carry their babies and care for them at night [34]. Some mothers with visual impairments belief in their capacity to breastfeed their newborns [35], and are assisted and motivated to breastfeed using assistive audio technology [36], which is a well-organized, systematic printed material with appropriate audio styles made up of novels, songs and poems to enable proper understanding of breastfeeding [36].

Studies have shown that the maternal health care of women with disability is compromised as a result of inadequate information and support from health care professionals during pregnancy, childbirth and post-delivery care [17, 37–39]. According to a qualitative study to explore the challenges women with disability face in accessing and using maternal healthcare services in Ghana, it was found that disability also prevents women from having equal access to quality and skilled maternal health services as a result of unfavorable physical infrastructure and transportation constraints to health care settings [40]. Maternal healthcare service utilization by women with disabilities is also influenced by the negative attitudes as well as inadequate skills and knowledge displayed by nurses and midwives [40–44]. There seem to be dearth of literature in the experiences of blind mothers in Ghana. Therefore, this study explores the motherhood experiences of blind mothers in the Greater Accra Region of Ghana.

## Methods

### Aim, design and setting

The study aimed at exploring the experiences of blind mothers as they navigated the process of motherhood. Qualitative exploratory descriptive design was used to conduct the study. This approach allowed comprehensive and detailed descriptions of the experiences of blind mothers [45]. Accra, the capital city of Ghana which is located in the Greater Accra Region was the setting for the study. According to the Ghana Statistical Service 2021 report on disability, in Greater Accra Region of Ghana, 5.8% of the population have difficulty in performing an activity [46]. Greater Accra Region of Ghana although records the least percentage of people living with disability in Ghana compared to the rest of regions in Ghana [46], it was suitably selected for the study because Accra is home to the biggest rehabilitation center for people living with disabilities. The rehabilitation center attracts Ghanaians across the length and breadth of the country, thereby allowing the inclusion of participants from all parts of the country and increasing the convenient access to participants by researchers.

### Sampling and data collection procedures

Purposive sampling method was used to select participants into the study. One-on-one individualized interviews were conducted for nineteen blind mothers who opted to participate in the study and met the inclusion criteria. The inclusion criteria were; any woman who was blind in both eyes, had biologically given birth in the last ten years and lived with that child. They should also be the ones who could read through the use of braille. After ethical clearance had been given for the conduct of the study, the researchers approached authorities of the Center for Rehabilitation of People Living with disabilities who permitted an initial engagement with blind mothers during a meeting where the study objectives were explained and information sheets were distributed to prospective participants. The researchers attended an existing group meeting through the Rehabilitation Center for people living with disabilities. The group meets on monthly basis to see to the welfare of the people living with disabilities. About 65 women living with disabilities attended the monthly meetings during the period of data collection and around 40 were mothers. Those who took part in the study were those who met the inclusion criteria that is; those who were mothers. All the participants were recruited from among the blind mothers who attended the monthly meetings at the Rehabilitation Center for people living with disabilities. Participants were contacted later to find out whether they wanted to participate in the study. Those who opted to participate were scheduled and interviewed at their own convenience, time and place. Each individualized interview lasted between 30 min and an hour. Permission was sought from participants for interviews to be recorded. A semi-structured interview guide which was pilot tested and developed based on the aim of the study was used to interview clients. Data saturation was achieved on the nineteenth participant where no new information was received.

### Data analysis

The data analysis method which was used to analyze the data was content analysis as described by Padget [47]. Data collection and analysis were done concurrently which allowed probing in subsequent interviews. The data was cleaned to get rid of all identifiable data. After that, the transcripts were read severally to have a deep understanding of participants’ perspectives. Coding was then done by ataching names and phrases to sentences that captured the meanings of those sentences. Similar codes were put together to form sub-themes and sub-themes were aggregated for major themes to emerge. The researchers met severally to analyse the emerging themes and sub-themes to ensure that, it reflected the true meanings of participants’ perspectives.

### Trustworthiness of the study

The same interview guide was used to interview all participants in the study. Some of the participants were contacted later during data transcription and analysis for clarification and member checking. Field notes which were taken during data collection were used as back-ups during data transcription to verify participants’ responses. Concurrent data collection and transcription ensured that, emerging themes and sub-themes were probed in subsequent interviews. Data were transcribed verbatim from audio to text. This ensured that, participants’ perspectives were not distorted.

### Ethical considerations

Ethical clearance was obtained from the Institutional Review Board of the Noguchi Memorial Institute for Medical Research (NMIMR-IRB CPN 038/16–17, FWA 00,001,824). A letter was written to seek permission from the Association of Persons Living with Disabilities. The information sheet and consent form were written in braille to enable participants to read and sign. All participants were given information about the study several days before they voluntarily opted to participate in the study. All participants signed consent forms before participating in the study. Participants were made to understand that, they could opt out of the study at any time without any consequences. Participants’ identities were kept intact by representing them with codes and cleaning of data to prevent the appearance of any identifiable data.

## Results

### Participants’ background

Nineteen (19) participants were involved in the study. All participants were represented with alpha numeric codes. Their ages ranged between 20 and 34 years. All participants were residents of the Greater Accra Region but they originated from all Regions of the country. Eight of the participants were single mothers whereas the rest were living with their spouses. Their educational backgrounds ranged from illiteracy to high school certificate. All the mothers were unemployed. Eleven (11) of the mothers were primiparous and the other eight (8) were multiparous. Fourteen (14) of them were Christians and the rest of the five (5) were muslims. All the mothers had children under the age of ten years. Participants’ demographic data is in Table 1.

**Table 1:** Demographic characteristics of participants

No	Code	Age (yrs)	Level of Education	Parity	No. of Children	Religion
1	BM1	34	Illiterate	primiparous	1	Christianity
2	BM2	22	Illiterate	primiparous	1	Christianity
3	BM3	27	JHS	multiparous	3	Muslim
4	BM4	32	JHS	primiparous	1	Muslim
5	BM5	30	JHS	primiparous	1	Muslim
6	BM6	31	JHS	primiparous	1	Christianity
7	BM7	35	JHS	primiparous	1	Christianity
8	BM8	20	JHS	multiparous	4	Christianity
9	BM9	31	Illiterate	multiparous	3	Christianity
10	BM10	29	JHS	multiparous	3	Christianity
11	BM11	29	Illiterate	primiparous	1	Christianity
12	BM12	20	JSS	primiparous	1	Christianity
13	BM13	32	JHS	primiparous	1	Christianity
14	BM14	23	Illiterate	multiparous	2	Muslim
15	BM15	28	Illiterate	multiparous	3	Muslim
16	BM16	25	Illiterate	multiparous	4	Christianity
17	BM17	30	Illiterate	multiparous	2	Christianity
18	BM18	29	Illiterate	primiparous	1	Christianity
19	BM19	31	Primary	primiparous	1	Christianity

*BM* Mothers living with disability who were recruited, *Numeric from 1-19 attached to BM* Number based on the sequence of recruitment, *Yrs* Years, *JHS* Junior High School

### Themes and sub-themes

Three main themes emerged from the data: mothering role and difficulties (sub-themes; feeding, disciplining and protection), mothering challenges (sub-themes; discrimination and prejudice, financial distress and psychological distress) and coping strategies (prayer, children and self-motivation). Summary of themes and subtemes have been summerised in Table 2 below:

**Table 2:** Themes and the sub themes

Themes	Sub-themes
Mothering role and difficulties	• Feeding
	• Disciplining
	• Protection
Mothering challenges	• Discrimination
	• Prejudice
	• Financial distress
	• Psychological distress
Coping strategies	• Prayer
	• Children
	• Self-motivation

### Mothering role and difficulties

This theme describes the experiences of blind mothers with regards to feeding, disciplining and protection.

### Feeding

Navigating the process of motherhood was generally reported to be difficult by the mothers although fulfilling and joyous when achieved. In terms of feeding, mothers decribed their experiences with all forms of feeding; breastfeeding, bottle feeding and spoon feeding. Most participants lamented that, they found it very difficult to feed their children. There were even narratives where some participants mistakenly chocked their children in the process of feeding;

*“I am unable to breastfeed well unless I get someone to attach the baby well to my breast. But when I am able tobreasfeed, I feel joy. I am always suffering from sore nipples due to my nability to attach baby correctly to the breast.” (BM7).*

*“Spoon feeding of my baby has been very difficult for me. I once mistakenly dropped food into my child’s nose since I could not locate my infant’s mouth properly thereby chocking my child in the process” (BM12).*

*“Bottle feeding my child in my situation is one of the most difficult roles I dread as a mother. I really struggle to feed the baby through the bottle. I have difficulty in locating the mouth properly”(BM2).*

On the other hand, there were a few participants who were very confident in their ability to feed their children. Some were able to locate the mouths of their infants with spoon without any help.

“*My breastfeeding role as a mother is one of the most joyous experiences. I am able to locate the mouth of my child without any help and my ability to perform this role is what gives me a sense of fulfilment and afirmation as a mother” (BM15)*

### Disciplining

In this sub-theme, participants described how they disciplined their children anytime they misbehaved. Some reasoned with the children anytime there were reports of disobedients. Some of the children listened to them when they reasoned with them but others were stuborn and therefore ignored their moyhers. Others used coporal punishment which were mostly unsuccessful.

*“I am able to reason with my six year old child anytime I hear her insulting someone or misbehaving. I appeal to her sense of reasoning and she is able to identify with it and stop misbehaving. But for my nine year old boy, it is really a challenge. He never listens to anything I say and since I have no eyes to chase him whenever he faulters, what can I do?” (BM3).*

*“ I use my cane unsuccesfully to threaten to beat them anytime they misbehave. I believe in coporal punishment when a child misbehaves but I cannot see them to be able to punish them when the need arises. (BM11).*

### Protection

This sub-theme describes the physical protection that is given to the children of the participants. Most of the participants narrated that, they spent most of their daytime on the streets with their children and therefore are unable to physically protect their children from danger.

*“It is very dangerous out here on the street and I am unable to protect my child from danger. The cars are always speeding and sometims my child walks on the street and I wish there was more I could do to protect him.”(BM13)*

*“My children are extremely vulnerable out here in the streets. There are times that I wish I could do more to protect my children from danger but there is nothing I can do. I have to rely on the benevolence of others for their protection.” (BM8)*

*“I am afraid for my child oo. Anytime he steps out of this house, my heart misses a beat. I feel that, he is in danger but I’m blind and therefore cannot protect him”(BM17)*

### Mothering challenges

In this theme, participants described challenges they faced as they navigated through the process of motherhood. They raised issues pertaining to discrimination and prejudice, financial distress and psychological distress.

### Discrimination and prejudice

All the participants felt discrriminated and pre-judged for being mothers because of their physical disability. Some participants said that, they felt discriminated anywhere they found themselves; whether in the hospital or any other public space. One of them was of the view that, constant prejudice makes her sometimes believe that, she is even not worthy of being a mother.

*“Once, I heard someone telling another person that ‘even this blind woman too has given birth. Why do they get themselves pregnant in the first place? It is very annoying to see them with children’ Tears immediately rushed down my cheeks when I heard it and that makes me wonder sometimes if I am worthy of motherhood” (BM19)*

*“Some people question my ability to be a good mother all the time. A neighbour once insulted me for giving birth. In her opinion, I was wicked to have given birth knowing that I was blind. I have heard that severally to an extent that, sometimes I tend to believe that, maybe, I’m not worthy of motherhood as a blind person” (BM4)*

*“I was once ignored by one nurse at a publuc hospital when I was in labour. She asked me sarcastically ‘who asked you to get pregnant when you know that you are blind? How can you take care of this child?’ I wept not because of the labour pain I was experiencing but I wept because of the way I was treated and spoken to”(BM13)*

One participant had a more positive outlook when it came to the issue of discrimination and prejudice.

*“As for me, I get a lot of previledges because I am a blind mother. Anywhere I find myself, people are willing to help me”BM7*

### Financial distress

Almost all the participants complained about their finances. According to them, money is difficult to come by. They wallow in poverty and that makes it difficult to make ends meet. They attribute this to their inability to secure jobs due to their perculiar circumstances.

*“I have a lot of financial difficulties. I am unable to make ends meet. I struggle to make any money since I cannot secure any job. I am a begger and not everyone has sympathy on mothers like us. Although I am on the streets with my children, I don’t get much financial help” (BM1).*

*“Poverty has gripped me and my children. I am always distressed due to financial problems. I struggle to buy food and other basic needs for my child and myself” (BM14). “I’m always in distress because of poverty. I hardly make any money because I cannot secure any job” (BM3)*

### Psychological distress

Almost all the participants complained about psychological distress. Participants complained of fear, anxiety and depression. Participants described how they wept most times coupled with feelings of anxiety and fear.

*“The challenges I am facing as a blind mother weigh so much on me to an extent that, I feel sad and depressed all the time. The sadness pushes me to cry most of the time. I easily get tears in my eyes when I think of my inability to provide for my child and protect her” (BM12).*

*“I’m anxious all the time since I became a mother. This gives me palpitations. I cry a lot when most times I cannot give my children a three square meal”(BM4).*

*“I’m afraid for myself and my children. Fear grips me all the time when I think of the future of my children. I wonder how the future is going to be for us” (BM3).*

### Coping strategies

Participants narrated that, they coped with their challenges with prayer, hope in their children and self-motivation. Majority of the participants encouraged themselves with the above mentioned means in order to survive and bear with the challenges of being a blind mother.

### Prayer

Prayer was the safe haven for most of the participants. Participants prayed to God to miraculously provide solutions to their problems. Prayer was done in such a way that, they could talk to God at different times.

*“Prayer is my safe haven oo. I sometimes pray myself to sleep in tears. I believe in God and I hope that, one day, he will open doors for me and my children” (BM3).*

*“Prayer is what has saved my life. Anytime I feel depressed, I pray to God fervently to help me out” (BM16)*

### Children

Participants were of the view that, their children were their source of hope. To them, the future held promising prospects so far as their children had the eyes to help them navigate their suroundings as they grew up.

*“My children are my hope and source of joy. They give me hope for the future and they are the reason why I am still alive”(BM7)*

*“Since my child turned seven years, he has been my source of solace and my eyes. He is the one who helps me to navigate my environment. He is my eyes and my source of hope” (BM9)*

*“I don’t know how I would have coped without my child. He is the reason why I am able to wake up every morning”(BM1).*

### Self-motivation

Few of the participants coped with their challenges by motivating themselves. To them, their best shot at survival was self-motivtion. Participants reported that, they felt that, the best person to push them was themselves and that was why they pushed themselves.

*“As for me, I encourage myself all the time. The best person to encourage me is me myself. The world feels so dark to me as a blind mother and therefore, if I don’t encourage myself I would be depressed all time” (BM14)*

*“I talk to myself all the time in order to self assure myself that, there may be light at the end of the tunnel” (BM2)*

*“Self motivation is what has saved my life. I used to be very sad all the time. My depression has reduced a bit since I started motivating myself” (BM8)*

## Discussion

The main findings of this study brought to bare the fact that, most of the blind mothers admitted their inability to play their mothering roles by themselves. They narrated their difficulties when it came to feeding, disciplining and protecting their children. Participants were of the view that, their cumstances make it difficult to perform their roles without any assistance. This has been echoed by other researchers where most blind mothers tried mostly to perform their roles but met challenges in performing those roles and therefore needed assistance/social support in order to perform those roles [34, 48]. On the other hand it has been reported that, some blind mothers have a high sense of breastfeeding self efficacy that makes it easier for them to breastfeed their children with no assistance [35]. The issue of most blind mothers’ inability to perform their roles may be attributed to their inability to easily navigate their environment properly. There is therefore the need for the society as a whole to be apt in providing the neccesary support to such mothers so that they can succesfully play their mothering roles. Assistive audio devises have been reported to improve breasfeeding among blind mothers [36]. This kind of devise can be procured for blind mothers through government initiatives to assist blind mothers to breastfeed successfully.

On the issue of challenges faced by blind mothers, a lot of their challenges were about discrimination, prejudice about their incapabilities of becoming mothers, financial difficulties and psychological stress. Mothers described how members of the society and even health professionals discriminated against them and prejudged them for being blind mothers. Prejudice and discrimination has been widely reported in literature when it comes to the issue of blind mothers [14, 15, 28, 31, 49, 50]. In addition, health workers have also been reported to express doubts in the ability and suitability of blind mothers as parents to an extent that sometimes, they even go further to suggest abortion to such mothers [26]. Perhaps the discrimination and prejudice experienced by the mothers in this current study may be due to the doubts a lot of people have about the ability of people living with physical disabilities to attend to activities of daily needs independently. These perceptions concerning prejudice on the suitability of blind women as parents in some African countries are mostly informed by partriachial ideologies in some societies [30]. Meanwhile, there are people living with disabilities and have adapted to being independent with little or no help [35]. Therefore, the society, especially health professionals should be trained on how to attend specially to people living with disabilities since some mothers in this study reported discrimination from health workers as well. This is because, it has been reported from the Northern part of Ghana that, discrimination and poor treatment of blind mothers at antenatal clinics resulted in poor antenatal clinic attendance [17]. Poverty as reported by the mothers who participated in this study has also been recently documented in other studies by people living with disabilities [51–53]. In the ghanaian context, people living with all forms of disabilities have reported that, the poverty alleviation schemes did very little to alleviate hardships on them [52]. Perhaps these schemes are not tailored to solve long term poverty issues but rather adhoc relief measures. Therefore, blind mothers and their children should be given the opportunities to access certain funds that can constantly relieve them from poverty and take them off the streets.

Blind mothers in this current study reported that, they coped with their challenges with prayer, self motivation and the hope they had in their children. With all the challenges, mothers with disabilities have been documented to motivate themselves to be resilient [54]. Feelings of gratifications about the ability of women with disabilities to be mothers have been reported to give them the urge to survive despite the challeges motherhood brings [28]. Perhaps the validation motherhood gives to blind mothers about the fact that, they too can experience the joys of motherhood is what explains their coping strategies. Such validations are neccesary to give a sense of worth and wellbeing. Therefore, the society should be sensitized on the need to embrace mothers with disabilities in general since they also deserve all the rights enjoyed by all humans including the right to reproduction.

### Limitation of the study

The main limitation of the study was the challenge of recruiting participants who could read the information sheet and consent form in braille. The sample size of the study reached upon data saturation was nineteen hence limited the generalizability of the study findings. The geographical location of the study was the Greater Accra Region of Ghana. However, the study could have included other mothers living with blindness from other geographical regions of Ghana to increase the diversity of the study sample and generate varied perspectives.

## Conclusion

It was apparent from this empirical study that, blind mothers who participated in this study had lots of challenges playing their roles in the areas of feeding, disciplining and protection. Most importantly, most of the mothers attested to the fact that, they found difficulties in protecting their young ones from danger. The mothers complained about challenges such as poverty due to their inability to work, discrimination and prejudices about the appropriateness of them being mothers and psychological stresses. In all their tribulations, they coped with prayers, self motivation and the hope they had in their children. It is the hope of the authors that, throwing light on this issue which has been largely negleted would divert more attention to the issues of blind mothers since they need help to navigate the process of motherhood.

Based on the results of the study, the recommendations below have been made as possible ways in which this research could contribute to the development of support, policies and interventions to overcome some of the challenges faced by these mothers.

1. The findings highlight the challenges that are experienced by blind mothers in obtaining assistive devices. Therefore, policy makers may learn from these findings to support blind mothers with assistive devises through governmental initaitives to help improve their breasfeeding practices.
2. Discrimination and prejudice featured promptly in the findings.This may trigger proactive interventions by health professionals to sensitize the society and health managers to train health professionals on skills to handle mothers living with diabilities.
3. Policy makers may learn from the findings to reinforce the poverty alleviation schemes which are aimed at offering the blind mothers and their children opportunities to access certain funds to support their daily up keep.
4. The Ministry of Gender, children and social protection of Ghana may learn from the findings to sensitize the general public to respect the rights of people living with disabilities especially their reproductive rights.

## Data Availability

The datasets analyzed during this current study are available from the corresponding author on reasonable request.
